# Persistence, Spatial Distribution and Implications for Progression Detection of Blind Parts of the Visual Field in Glaucoma: A Clinical Cohort Study

**DOI:** 10.1371/journal.pone.0041211

**Published:** 2012-07-27

**Authors:** Francisco G. Junoy Montolio, Christiaan Wesselink, Nomdo M. Jansonius

**Affiliations:** 1 Dept. of Ophthalmology, University Medical Center Groningen, University of Groningen, Groningen, The Netherlands; 2 Dept. of Epidemiology, Erasmus Medical Center, Rotterdam, The Netherlands; Univeristy of Melbourne, Australia

## Abstract

**Background:**

Visual field testing is an essential part of glaucoma care. It is hampered by variability related to the disease itself, response errors and fatigue. In glaucoma, blind parts of the visual field contribute to the diagnosis but - once established – not to progression detection; they only increase testing time. The aims of this study were to describe the persistence and spatial distribution of blind test locations in standard automated perimetry in glaucoma and to explore how the omission of presumed blind test locations would affect progression detection.

**Methodology/Principal Findings:**

Data from 221 eyes of 221 patients from a cohort study with the Humphrey Field Analyzer with 30–2 grid were used. Patients were stratified according to baseline mean deviation (MD) in six strata of 5 dB width each. For one, two, three and four consecutive <0 dB sensitivities in the same test location in a series of baseline tests, the median probabilities to observe <0 dB again in the concerning test location in a follow-up test were 76, 86, 88 and 90%, respectively. For <10 dB, the probabilities were 88, 95, 97 and 98%, respectively. Median (interquartile range) percentages of test locations with three consecutive <0 dB sensitivities were 0(0–0), 0(0–2), 4(0–9), 17(8–27), 27(20–40) and 60(50–70)% for the six MD strata. Similar percentages were found for a subset of test locations within 10 degree eccentricity (P>0.1 for all strata). Omitting test locations with three consecutive <0 dB sensitivities at baseline did not affect the performance of the MD-based Nonparametric Progression Analysis progression detection algorithm.

**Conclusions/Significance:**

Test locations that have been shown to be reproducibly blind tend to display a reasonable blindness persistence and do no longer contribute to progression detection. There is no clinically useful universal MD cut-off value beyond which testing can be limited to 10 degree eccentricity.

## Introduction

Glaucoma is a progressive disease that may cause irreversible blindness. Monitoring of the disease with perimetry is an essential part of glaucoma care, unless patients have a short life expectancy and little glaucomatous damage. Variability hampers the use of perimetry in detecting small changes in visual function. In glaucoma, variability is presumably related to response errors, fatigue effects [1], [2] and a flatter frequency-of-seeing curve in regions with a reduced sensitivity [3], [4]. The development of the Swedish Interactive Threshold Algorithm (SITA) strategies for the Humphrey Field Analyzer (HFA) has partially resolved the fatigue issue by reducing the test time [5].

SITA reduces the test time, amongst others, by predicting the sensitivity in a test location from the sensitivity in neighboring test locations and by incorporating general knowledge on glaucomatous visual field patterns. However, SITA ignores an obvious other source of prior knowledge, being the previous test result. The use of the previous test result can reduce test time [6], [7] and test-retest variability [8]. To illustrate this, for a typical glaucomatous visual field, that is, a blind superior hemifield together with an intact inferior hemifield, the test time of SITA is about 1.5 times longer than for a normal field. Hence, to establish blindness in a test location takes twice as long as establishing a normal sensitivity – and thus a 33% test-time reduction should be possible by incorporating information from previous tests. This is in agreement with earlier findings [7]. To go one step further, if the superior hemifield would have been unresponsive on several consecutive occasions, it makes no sense to test it again: only the inferior hemifield needs to be tested to monitor the eye. Hence, a 67% test-time reduction would ultimately be possible in this case.

**Table 1:** Example of two patients as represented in the database, with two and eight test locations with a sensitivity of <0 dB in the fourth visual field, respectively.

Patient	VF1(dB)	VF2(dB)	VF3(dB)	VF4(dB)	VF5(dB)	VF6(dB)	VF7(dB)	VF8(dB)	Position on VF	% <0 dB	Mean (dB)
1	4	4	6	<0	0	0	0	11	4	0	2.75
1	<0	<0	<0	<0	<0	2	<0	18	9	50	4.00
2	12	3	11	<0	<0	<0	13	10	1	50	4.75
2	20	0	5	<0	<0	<0	9	0	2	50	1.25
2	<0	<0	<0	<0	<0	3	<0	<0	19	75	−0.75
2	15	12	<0	<0	11	<0	<0	4	20	50	2.75
2	20	2	<0	<0	<0	1	<0	<0	30	75	−1.25
2	21	<0	6	<0	16	<0	<0	4	31	50	4.00
2	26	12	4	<0	10	4	5	0	33	0	4.75
2	<0	3	<0	<0	<0	10	<0	<0	35	75	1.00

VF  =  visual field; columns VF1-VF4 refer to baseline, VF5-VF8 to follow-up; last two columns depict the data analysis as applied to the follow-up data (for details see text).

**Table 2:** Patient characteristics for all included 221 eyes of 221 patients and for the subset of 53 eyes of 53 patients with at least eight visual field tests and at least one test location showing a <0 dB sensitivity on four consecutive baseline tests (mean with standard deviation between brackets unless stated otherwise).

	N = 221	N = 53
**Baseline**		
Age (years)	65.1 (12.3)	65.1 (10.3)
Gender (% male)	55.2	45.3
Right eye (%)	50.7	52.8
Mean Deviation (median [interquartile range]; dB)	−7.0 (−14.5 to −3.0)	−14.7 (−18.7 to −10.9)
**Follow-up**		
Follow-up duration (years)	6.4 (1.2)	6.9 (1.0)
Rate of progression (median [interquartile range]; dB/year)	−0.1 (−0.5 to +0.1)	−0.2 (−0.5 to 0.0)
Square root of the residual mean square of Mean Deviation (dB)	1.1 (0.7)	1.2 (0.7)

The aims of this study were (1) to describe the persistence and spatial distribution of blind test locations in standard automated perimetry in glaucoma and (2) to explore how the omission of presumed blind test locations would affect progression detection. For the first aim, we determined the probability to observe a sensitivity below a certain value as a function of the number of preceding consecutive sensitivities below that value in the concerning test location. This was evaluated for <0, <5, <10 and <20 dB. The value <0 dB corresponds to the maximum stimulus intensity of the HFA perimeter; the values <5 and <10 dB approximately to the maximum stimulus intensities of the Octopus and Oculus perimeters, respectively. Subsequently, we compared the percentages of blind test locations between the regular standard automated perimetry 30–2 grid (with test locations up to 30 degree eccentricity) and the subset of test locations falling within the 10–2 grid (up to 10 degree eccentricity), as a function of disease stage as defined by the mean deviation (MD). The aim here was to determine a clinically useful MD cut-off value for preferring 10–2 testing over 30–2 testing in advanced glaucoma. After all, although glaucoma sometimes starts close to fixation [9], [10], it is conceptually a disease affecting the peripheral visual field first and thus a transition from 30–2 to 10–2 testing would be the easiest way to avoid uninformative testing of unresponsive parts of the visual field in advanced disease. For the second aim, we studied the performance of an MD-based progression detection algorithm with and without assuming blind test locations as established at baseline to be blind in all follow-up fields.

**Figure 1:**
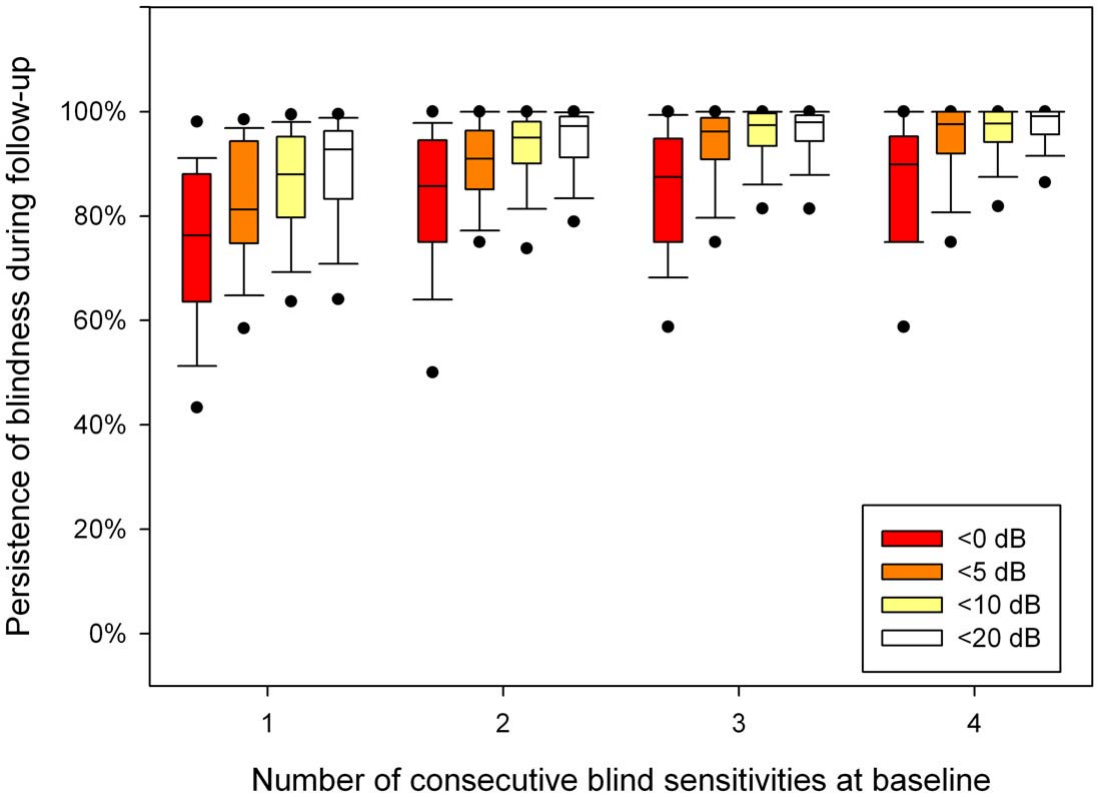
Percentage of follow-up sensitivities being <0, <5, <10 and <20 dB as a function of the number of consecutive <0, <5, <10 and <20 dB baseline sensitivities. Boxplots show median, interquartile range, and 5^th^, 10^th^, 90^th^ and 95^th^ percentiles.

**Figure 2:**
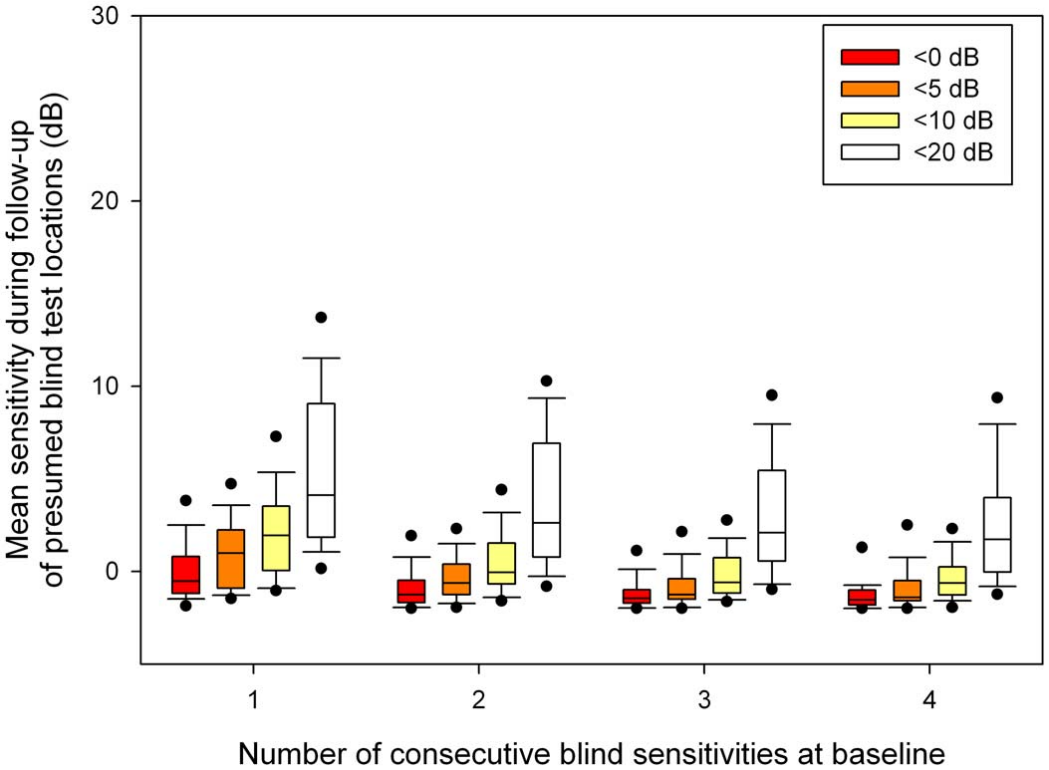
Mean sensitivity during follow-up in test locations with 1, 2, 3 and 4 consecutive <0, <5, <10 and <20 dB baseline sensitivities. Boxplots show median, interquartile range, and 5^th^, 10^th^, 90^th^ and 95^th^ percentiles.

**Table 3:** Analysis of variance with the number of consecutive tests showing blindness in a test location (N) and the definition of ‘perimetrically blind’ (B) as within-subject factors and the persistence of blindness as dependent variable.

	df	MS	dfe	MSe	F	P
mean	1	680.65	52	0.08460	8045	<0.001
N	3	0.47354	156	0.00702	67	<0.001
B	3	0.57498	156	0.01089	53	<0.001
N*B	9	0.00547	468	0.00161	3	<0.001

df  =  degrees of freedom; MS = mean squares (MS = SS/df with SS = sum of squares); dfe is df for error; MSe = mean squares for error**.**

## Methods

### Ethics Statement

The study protocol was approved by the ethics board of the University Medical Center Groningen. This board approved that for the current study no informed consent had to be obtained because the study comprised a retrospective anonymous analysis of visual field data collected during regular glaucoma care. To ensure a proper glaucoma diagnosis of the included patients, we limited the study population of this study to glaucoma patients that had been included in the Groningen Longitudinal Glaucoma Study (GLGS) in the past. In the GLGS, all glaucoma patients and glaucoma suspects who visited the glaucoma outpatient service of the University Medical Center Groningen between July 1, 2000, and June 30, 2001, and who provided informed consent were included in an observational study with conventional perimetry, frequency-doubling perimetry (FDT; Carl Zeiss Meditec AG, Jena, Germany) and laser polarimetry (GDx; Laser Diagnostic Technologies, San Diego, California, USA). Patients received written information at home at least two weeks before their regular care visit that was flagged as the baseline visit of the study. The receipt of the information and agreement to participate was checked verbally during the concerning visit. The aim of the study was explained; participation was voluntary and participation could be stopped also after having agreed to participate. The study essentially comprised the collection of regular care data obtained during regular visits and an additional FDT and GDx test embedded in a regular visit. FDT and GDx are non-invasive diagnostic tests with a very limited additional burden and no additional risk for the patient. The protocol of the original GLGS was approved by the department of Medical Technology Assessment of the University of Groningen. The original health technology assessment research question was if it was possible to replace, in glaucoma patients and/or glaucoma suspects, the lengthy and cumbersome conventional perimetry by FDT and/or GDx. The study followed the tenets of the Declaration of Helsinki.

**Figure 3:**
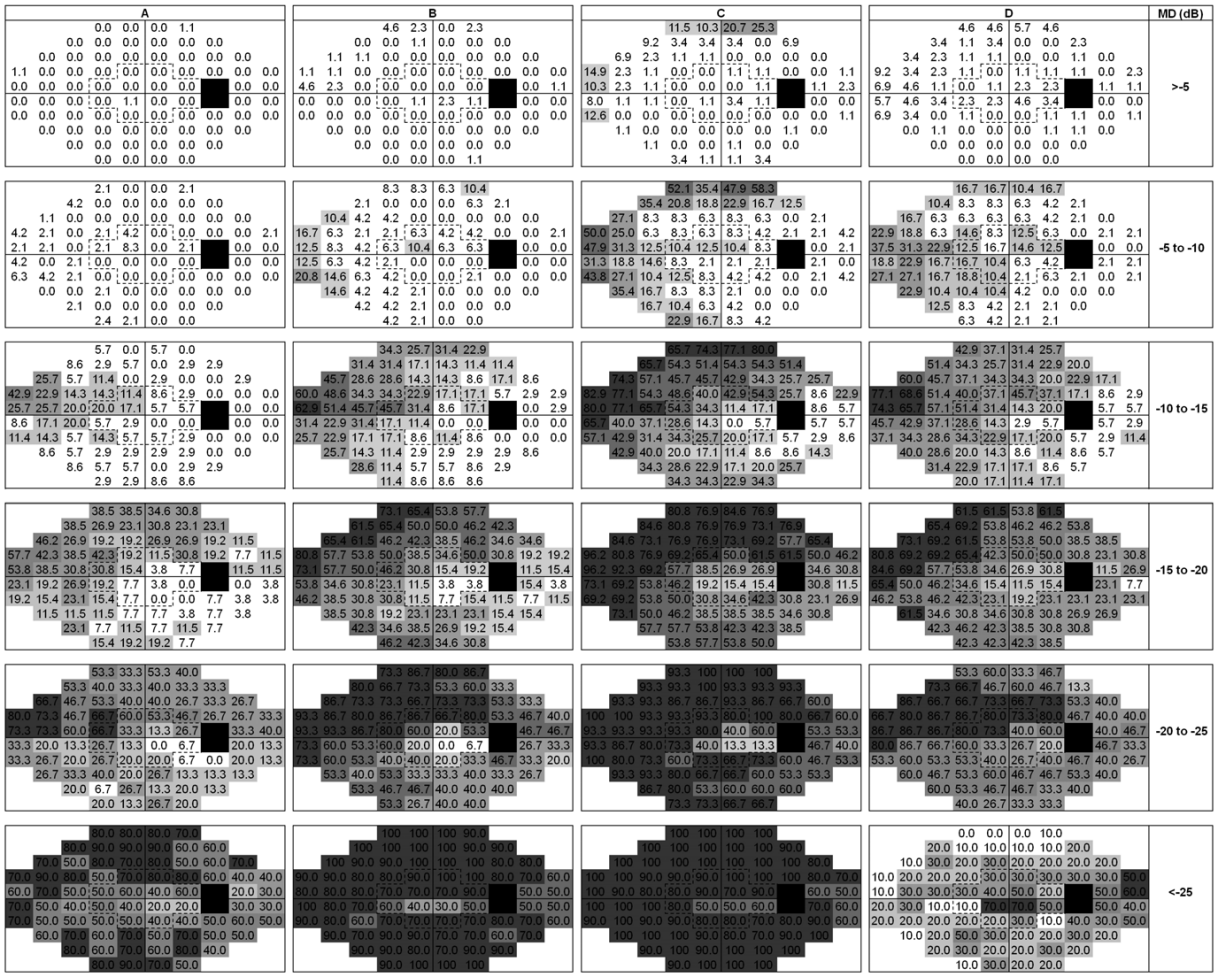
Spatial distributions of test locations that met various criteria for blindness, as a function of baseline mean deviation (MD). The criteria were three consecutive sensitivities of <0 (A), <10 (B) and <20 (C) dB, and being ‘out of range’ according to the Glaucoma Progression Analysis (GPA; D). Black squares are the blind spot; white squares are test locations flagged as blind in 0–10% of the patients. The remaining intermediate four gray scales denote, from light to dark, blindness in 10–20%, 20–40%, 40–60% and above 60% of the patients.

### Study Population

Details of the GLGS have been described earlier [11], [12]. In short, after the initial health technology assessment study described above, we continued performing conventional perimetry in glaucoma patients and moved to FDT/GDx in glaucoma suspects in our regular care. The GLGS continued as an ongoing anonymous gathering of all information from glaucoma patients and glaucoma suspects obtained during regular care. For the present study, we used data from a subpopulation of the GLGS cohort: patients had to have (1) glaucoma at baseline (for criteria see below) and (2) at least four (five with discarded learning test) standard automated perimetry tests (HFA; Carl Zeiss Meditec Inc., Dublin, CA).

**Figure 4:**
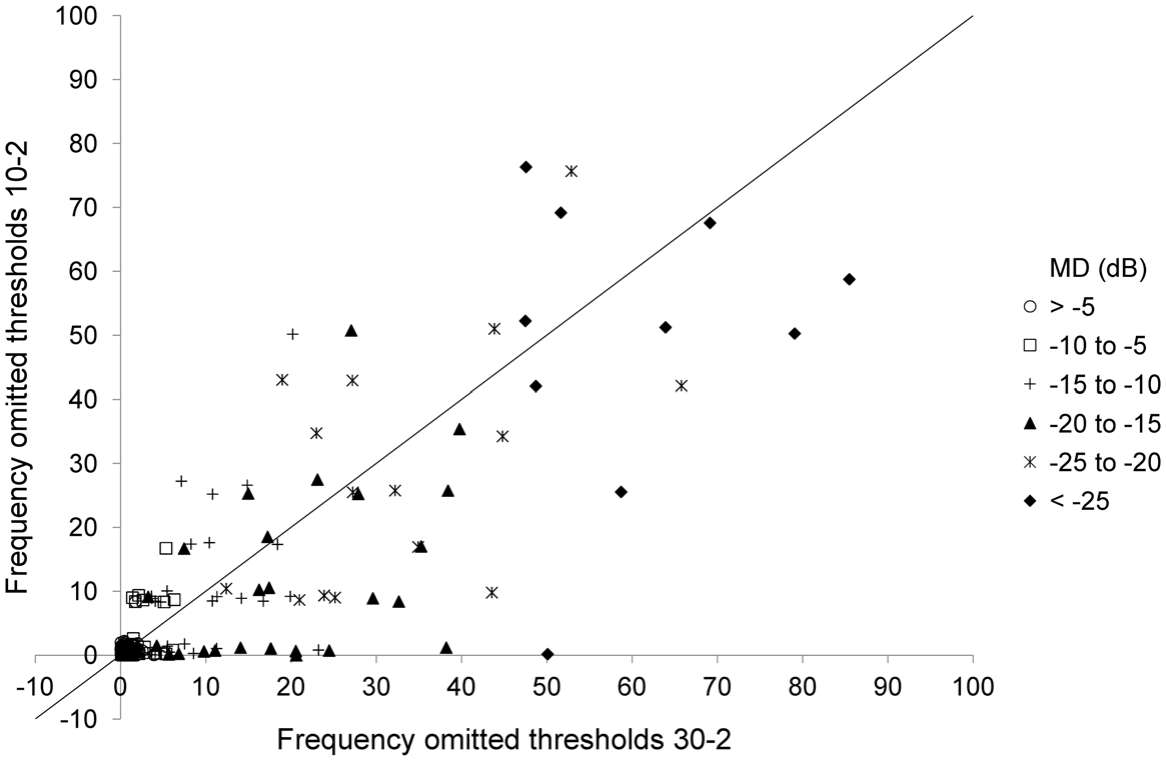
Percentage of blind test locations according to the three consecutive <0 dB criterion for all test locations within the 30–2 grid (x-axis) versus a subset of test locations within the 10–2 grid (y-axis). Symbols indicate stratification according to baseline mean deviation (MD) in six strata, being up to -5 dB, from -5 to -10 dB, -10 to -15 dB, -15 to -20 dB, -20 to -25 dB en beyond -25 dB. Noise with a standard deviation of 1% was added in order to avoid overlapping data points.

### Perimetry

Perimetry was performed using the HFA 30–2 SITA fast strategy. For glaucoma, two consecutive, reliable tests had to have defects according to previously published criteria [11], [12]. For being reproducible, defects had to be in the same hemifield and at least one depressed test point of these defects had to have exactly the same location on both tests. Moreover, defects had to be compatible with glaucoma and without any other explanation (for example, cataract, macular degeneration or lesions of the central visual pathways). Prior to these two tests, another test had to be made and this test was excluded to reduce the influence of learning. During the follow-up period, perimetry was performed at a frequency of one test per year. In case of suspected progression or unreliable test results, clinicians could increase the frequency of testing. This was a subjective decision; no formal tools or rules were given (observational study design).

**Figure 5:**
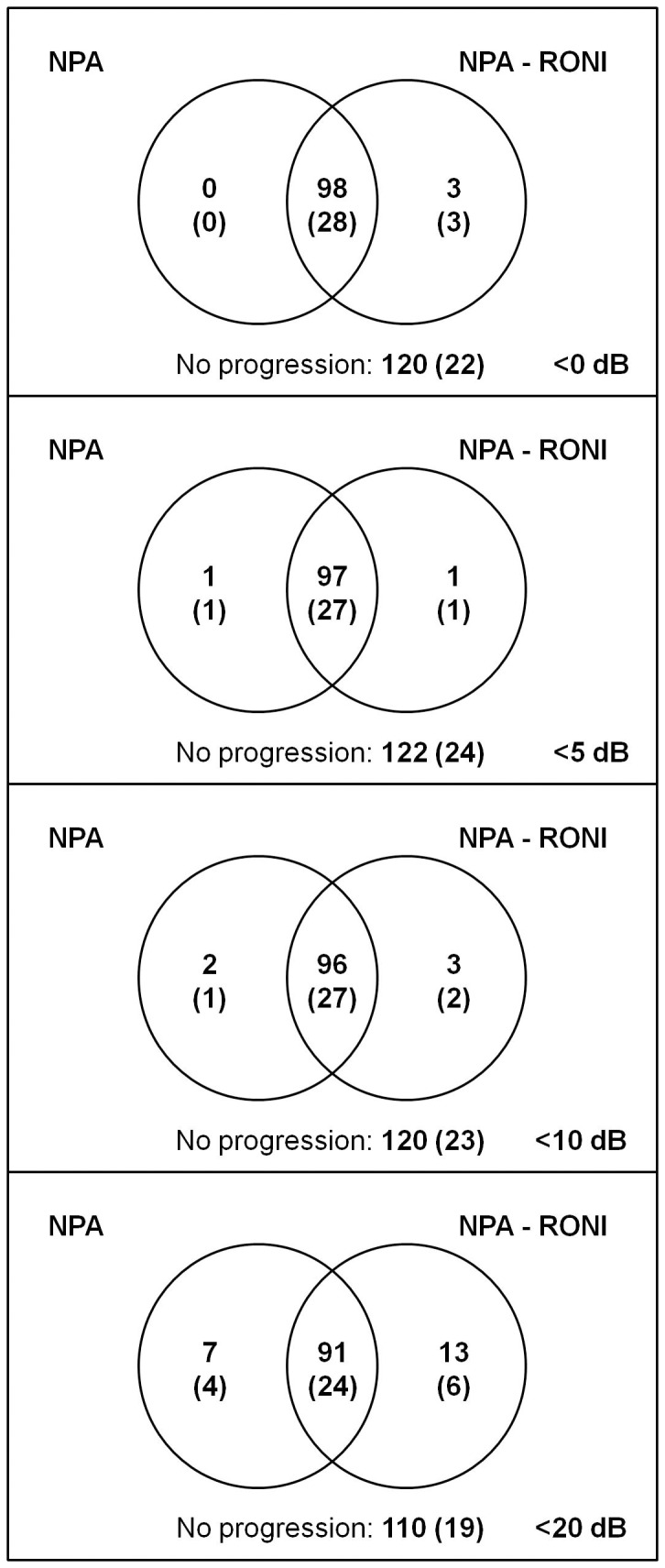
Venn diagrams showing progression according Nonparametric Progression Analysis (NPA) versus NPA after removing all test locations that were blind on the first three tests (NPA-RONI, where RONI is regions of no interest). Four different definitions of blindness were used: <0, <5, <10 and <20 dB. Results for all 221 subjects with results for the subset of 53 subjects between brackets.

### Data analysis

One eye per patient was included. If both eyes met the above-described criteria, one eye was chosen randomly. For anatomical representation, all left-eye threshold data were converted to a right-eye format. Thresholds representing the blind-spot were excluded from the analysis, leaving 74 tests locations for analysis.

**Figure 6:**
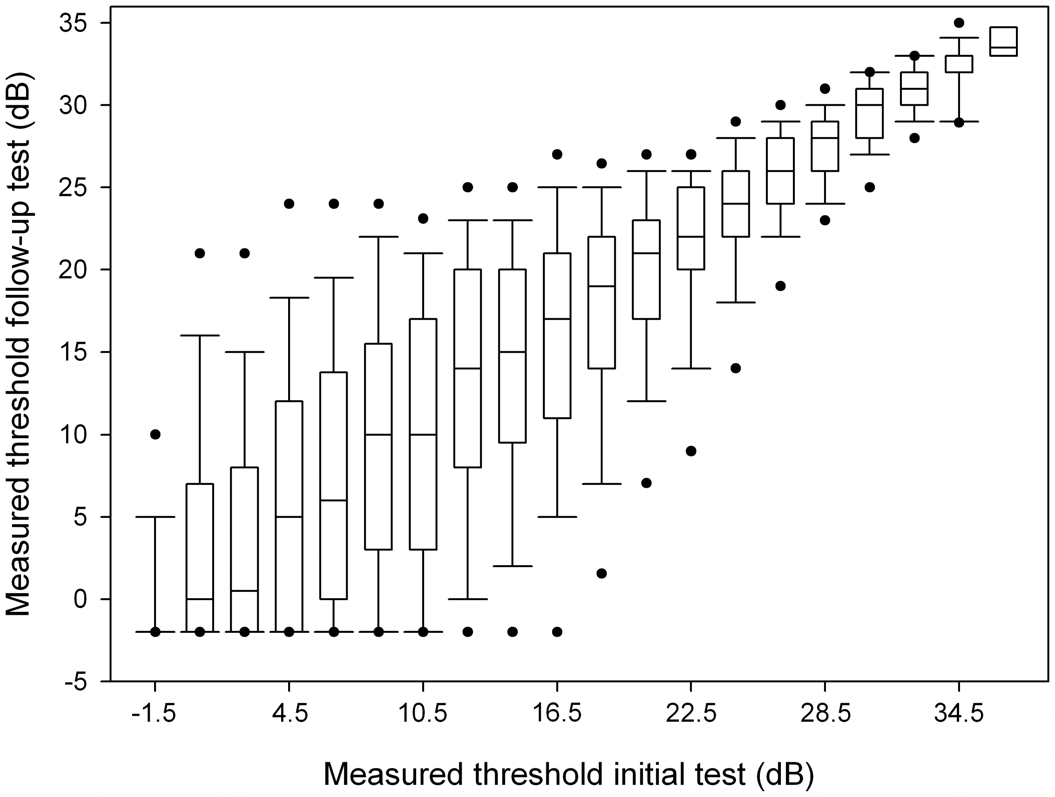
Pointwise test-retest variability. Data presented in strata of 2 dB, except for <0 dB which was set to -1.5 dB in one box. Boxplots show median, interquartile range, and 5^th^, 10^th^, 90^th^ and 95^th^ percentiles.

#### Persistence of blindness

For this analysis we only included patients who (1) performed at least eight tests and (2) had at least one test location showing a <0 dB sensitivity on four consecutive baseline tests (most stringent criterion for blindness). We defined four subgroups of test locations, based on the first four tests and named VF4<0, VF3-4<0, VF2-4<0 and VF1-4<0. A test location VF4<0 had to have a sensitivity of <0 dB in the fourth visual field test. A test location VF3-4<0 had to have a sensitivity of <0 dB in both the third and the fourth test, and so on. For VF4<0, the sensitivity of the test location in the third test may or may not be <0 dB. Hence, VF3-4<0 is a subset of VF4<0, and so on. We took the fourth test as a reference in order to be able to vary the number of baseline tests without the need of changing the selection of the four follow-up tests, which were the fifth to eighth test.

For all test locations with a sensitivity of <0 dB in the fourth test, we analyzed the corresponding sensitivities in the four follow-up tests. Outcome measures were (1) the percentage of follow-up tests showing a sensitivity of <0 dB and (2) the mean sensitivity. Here, test locations with <0 dB were set at -2 dB. This is the arbitrary interpretation of <0 dB as chosen by the manufacturer. For patients, the difference between 0 dB and <0 dB implies seeing the maximum light stimulus of the perimeter (0 dB) or not (<0 dB).

Test locations within a single subject cannot be considered independent. Therefore, to avoid that a few patients with many blind test locations would dominate the results, we first determined the averages and corresponding standard deviations of the outcome measures within each patient for each subgroup of test locations (VF4<0, VF3-4<0, VF2-4<0 and VF1-4<0). Subsequently, the averages were presented using nonparametric descriptive statistics and the standard deviations of the first outcome measure were averaged over all patients and presented as the “mean within-patient standard deviation”.

Table 1 gives an example of two patients as represented in the database. These patients are present in the VF4<0 subgroup with two and eight test locations, respectively. The first patient is also present in the VF3-4<0, VF2-4<0 and VF1-4<0 subgroups, with one test location. The second patient is present in these subgroups with four, one and one test locations, respectively. For the first patient, blindness persistence was 25% for the VF4<0 subgroup and 50% for the VF3-4<0, VF2-4<0 and VF1-4<0 subgroups. For the second patient, this was 53, 69, 75 and 75% for the VF4<0, VF3-4<0, VF2-4<0 and VF1-4<0 subgroups, respectively.

The analyzes were repeated with blindness of a test location defined as a sensitivity of <5, <10 and <20 dB instead of <0 dB. The influence of the number of consecutive tests (1, 2, 3 or 4) showing blindness in a test location and the definition of blindness (<0, <5, <10 or <20 dB) on the persistence of blindness was analyzed with ANOVA, with the persistence of blindness (average percentage of follow-up tests showing blindness in the concerning test locations) as the dependent variable.

#### Spatial distribution of perimetrically blind test locations

For this analysis, we included all patients who performed at least four tests. Patients were stratified according to baseline MD in six strata, being above -5 dB, from -5 to -10 dB, -10 to -15 dB, -15 to -20 dB, -20 to -25 dB en beyond -25 dB. We plotted the test locations considered blind based on their sensitivity history and calculated the percentages of these test locations, for all test locations of the 30-2 grid and for a subset laying within the 10-2 area. Percentages were compared with a nonparametric paired test (Wilcoxon).

A commonly used progression detection algorithm, the Glaucoma Progression Analysis (GPA) [13], has its own built-in criterion for blindness: a cross on the printout indicates that the test location is ‘out of range’ and not used for progression detection by the software. We compared – for all six MD strata - the spatial distributions and percentages of test locations flagged as ‘out of range’ by GPA with that of test locations considered blind based on their sensitivity history. Percentages were compared using a nonparametric paired test (Wilcoxon).

#### Influence of assuming blindness on progression detection

If the sensitivity of a test location has been below a certain value on a number of consecutive tests, it might be an efficient approach to consider such a test location blind in all future tests – in glaucoma – without actually retesting it. This might result in a (slight) underestimation of the MD and thus might affect MD-based progression detection algorithms. To determine the influence of this approach on clinical decision making, we classified all included eyes as stable or progressing according to the MD-based Non-parametric Progression Analysis algorithm (NPA), with progression defined as at least possible progression at the end of the follow-up (NPA is based on a nonparametric ranking of MD values; for possible progression, the MDs of the last two tests have to be lower than the lower MD of two baseline tests) [12]. Subsequently, we repeated this after assuming test locations to be perimetrically blind based on their sensitivity history. Here, we excluded test locations from the analysis if they were blind on the first three tests, according to four different definitions of blindness: <0, <5, <10 and <20 dB. For all four definitions, both classifications were compared with a McNemar test. Because the MD is an average weighed to test location eccentricity, and the weigh factors are unpublished, we applied the NPA criterion to the eccentricity-uncorrected average sensitivity of all test locations (mean sensitivity).

Calculations and statistical analyses were performed using SPSS Statistics 18.0 (SPSS Inc., Chicago, IL); the ANOVA was performed using MrF (http://psy.otago.ac.nz/miller/).

## Results

Table 2 shows the patient characteristics. Two-hundred-twenty-one patients were included of which 53 performed at least eight tests and had at least one test location showing a <0 dB sensitivity on four consecutive baseline tests. The average follow-up durations were 6.4 and 6.9 years, respectively, with median MD values at baseline of −7.0 and −14.7 dB.

Figure 1 shows the blindness persistence characteristics as a function of the number of consecutive baseline sensitivities below <0, <5, <10 and <20 dB. The boxplots visualize the between-patient variability; the corresponding mean within-patient standard deviations were, following the sequence of Fig. 1 from left to right, 25, 23, 21, 20, 16, 15, 13, 12, 14, 10, 9, 10, 13, 9, 9 and 8%. If the number of consecutive baseline tests on which a test location was blind increased, the probability of being blind during follow-up increased. The increase in blindness persistence appeared to saturate at three consecutive baseline sensitivities below the concerning value. Blindness persistence appeared to be highest for <10 and <20 dB and lowest for <0 dB. Table 3 shows that blindness persistence depended significantly on both the number of consecutive tests showing blindness in a test location (P<0.001) and the definition of blindness (P<0.001). Figure 2 presents the corresponding mean sensitivity as recorded during the four follow-up tests in the presumed blind test locations.

Figure 3 illustrates the spatial distributions of test locations that met the requirement of three consecutive sensitivities of <0 (A), <10 (B) and <20 (C) dB, and that were ‘out of range’ according to the GPA (D), as a function of baseline MD. The number of blind test locations increased monotonically with MD for all criteria of blindness except for GPA; for GPA the number of test location flagged as ‘out of range’ decreased again with advanced glaucoma. As a consequence, significantly less sensitivities were ‘out of range’ according to GPA compared to blindness at <0 dB for baseline MD values below −25 dB *(P*<0.001), while the opposite was the case for all other strata (*P*<0.001). For the three consecutive <0 dB criterion (Figure 3A), the median (interquartile range) percentages of blind test locations were 0(0–0), 0(0–2), 4(0–9), 17(8–27), 27(20–40) and 60(50–70)% for the six MD strata.

Figure 4 presents a scatter plot showing the percentages of blind test locations according to the three consecutive <0 dB criterion for all test locations of the 30–2 grid versus a subset of 12 test locations located within the 10–2 grid. For the subset, the median (interquartile range) percentages of blind test locations were 0(0–0), 0(0–2), 6(4–11), 8(4–18), 23(13–48) and 50(40–70)% for the six MD strata. These percentages were similar to the corresponding percentages for the 30–2 grid (listed above) for all six MD strata (P = 0.32, 0.34, 0.11, 0.44, 0.17 and 0.23, respectively).

Figure 5 shows Venn diagrams indicating the number of eyes with at least possible progression at the end of the follow-up according to NPA versus NPA after removing all test locations that were blind on the first three tests (NPA-RONI, where RONI is regions of no interest) for four different definitions of blindness: <0, <5, <10 and <20 dB. There was no significant difference between the classifications by both approaches (P = 0.25, P = 1.0, P = 1.0 and P = 0.26 for <0, <5, <10 and <20 dB, respectively). Similar findings were done in the subset of 53 eyes (P = 0.25, P = 1.0, P = 1.0 and P = 0.75 for <0, <5, <10 and <20 dB, respectively).

## Discussion

Test locations with a sensitivity below a certain value on three consecutive occasions are unlikely to show a substantially higher sensitivity later on. Hence, if the concerning value corresponds to the maximum stimulus intensity of the perimeter used, these test locations do no longer contribute to progression detection. Omitting these locations from future tests will result in time saving without hampering progression detection. Obviously, the number of blind test locations (and thus the potential time saving) increases with increasing disease severity. Interestingly, the percentages of blind test locations appeared to be similar for 30–2 and 10–2 grids for all disease stages.

With the introduction of the SITA strategies in the late ninety’s of the previous century, the examination time of standard automated perimetry decreased substantially [14]. Unfortunately, this advantage over the full-threshold strategy is largely lost in severe glaucoma. Older Octopus strategies and the German Adaptive Threshold Estimation (GATE) algorithm overcome this increase in test time by using information from previous test results to determine more appropriate starting values for the stair-case procedure [6], [7]. We would suggest a further step by entirely omitting test locations that were shown to be blind at earlier occasions (‘regions of no interest’). This enables more time saving but obviously limits the application of our approach to irreversible eye diseases. Leaving out test locations may seem crude, but this is what is actually done by clinicians who exchange the default 30–2 grid by a 10–2 grid in advanced glaucoma and by clinicians who rely on GPA for progression detection. Interestingly, GPA ignores even more test locations than we propose to do with our ‘regions of no interest’ approach (see below and Results section). As GPA leaves them out in the analysis phase only, however, no time saving is obtained.

The time gained by the suggested approach should be interpreted and weighed correctly. Obviously, if the time saving is compared to the total time spent in the hospital, the saving is negligible. However, not testing blind test locations refrains a patient with moderate or advanced glaucoma from long time periods in which he or she does not observe any stimulus but has to stay alert nonetheless. This should increase concentration, thus increasing the reliability of the test result. Second, long time periods without any visible stimulus increase patient frustration by emphasizing not seeing things. Third, the saved time can be used to study the remaining parts of the visual field in more detail without additional visits or costs. This can be done by either adding test locations or determining thresholds more accurately. Obviously, to allow for a reliable progression detection throughout the follow-up, only the test locations belonging to the original grid should contribute to the MD. The added test locations, however, may be analyzed separately and may yield important information [9], [10].

A caveat of incorporating our regions-of-no-interest approach is that it may cause propagation of blindness through the visual field if applied to strategies that use some form of spatial smoothing (that is, do not determine a formal threshold in all individual test locations) in order to reduce test time (as possibly occurs in SITA). This will not occur in strategies that use neighboring sensitivities only for estimating a starting value for determining a threshold.

The classical picture of glaucoma deterioration is the development of visual field defects initially in the periphery, leaving vision unaltered centrally until the latest stages of the disease. Albeit this picture has been challenged recently [9], [10], the clinical translation of this picture is starting with 30–2 testing with a transition to 10–2 somewhere along the line - the easiest way to get rid of unresponsive parts of the visual field in advanced disease. One of the aims of this study was to develop a clinically useful guideline, that is, an MD cut-off value, for preferring 10–2 testing over 30–2 testing in advanced glaucoma. Interestingly, no such an MD value appeared to exist – the median percentage of blind test locations was essentially identical for 30–2 and 10–2 grids for all disease stages. With a closer look at our data, this corresponded to the three clinically well known patterns of visual field loss in severe glaucoma: (1) a central island without a peripheral (temporal) island, (2) a temporal island without a central island, and (3) both a central and a temporal island. This is also visible in Figure 4. Hence, in many patients a transition from 30–2 to 10–2 testing will never become an meaningful change. It is important to realize that we did not actually measure a 10–2 grid – a form of high spatial resolution perimetry [15], [16] - but analyzed a subset of 30–2 test locations laying within the 10–2 area. Here, the assumption is that this can be considered a representative (unbiased) sample. Also, inclusion of a patient in this study implied the presence of 30–2 fields. This might have induced a selection bias, as patients with only a central island might be underrepresented because they were at baseline already monitored with 10–2 testing – and thus excluded. This is unlikely, however, as at the baseline of the GLGS Goldmann perimetry and not 10–2 testing was the default escape in advanced glaucoma [11] – suggesting an underrepresentation of temporal islands rather than of central islands in this study. To conclude, the transition from 30–2 to 10–2 testing should be individualized and the advantage of a more detailed monitoring of a central island should be weighed against the need of building a new baseline and the loss of monitoring of any peripheral island. After all, it is not unlikely that progression in the periphery predicts future central loss.

Figure 3A-C actually depicts the “average” glaucoma progression pattern. Not unexpectedly, the glaucomatous deterioration starts nasal-superiorly. In agreement with the findings discussed in the paragraph above, both a central and a temporal island survived until the last MD stratum. With GPA, the number of test locations with a cross (indicating that the software ignores these location for progression detection) increases with disease progression up to an MD of about -20 dB but decreases beyond that point (Figure 3D). Although this pattern is identical to what is observed in the pattern deviation plot and is in agreement with the idea that GPA is based on pattern deviation analysis [17], it might mislead the clinician as it suggests erroneously that test locations that are actually blind are still monitored.

The absence of a response to the maximum stimulus intensity is not identical to blindness. The dynamic range of the perimeter can be increased by replacing stimulus size III by size V. Interestingly, this appears to reduce the test-retest variability [18]–[20]. Until now, however, the time-saving SITA strategy is not available for size V. Within a given stimulus size, it is not self-evidently beneficial to increase the dynamic range by increasing the maximum stimulus intensity. Although the well-known pointwise test-retest variability plot (for our data shown in Figure 6) suggests a reduced variability close to the maximum stimulus intensity, this is merely a floor effect. If we look in an alternative way to the same data (Figure 1), it might be the case that the extended dynamic range as used in HFA compared to Octopus and Oculus corresponds to a reduced reproducibility of blindness (Table 3). This is in line with the idea that a high test-retest variability is related to ganglion cell saturation [21], but requires further study.

The exclusion of test locations with a sensitivity of <0, <5 or <10 dB at baseline did not affect progression detection with NPA (Figure 5). Only for <20 dB some difference (albeit statistically not significant) appeared to occur. Here, progression according to NPA but not according to NPA-RONI might reflect deepening of existing defects (that is, test locations with a sensitivity already <20 dB at baseline); progression according to NPA-RONI but not according to NPA might be caused by a reduced variability in the calculated mean sensitivity for the RONI approach, which results in an increase in NPA sensitivity [22], [23]. These observations are in line with the findings described in the previous paragraph. It might be possible that other progression detection algorithms would be affected differently. This requires further study.

Originally, the SITA fast strategy, as used in the GLGS, was considered a time-saving improvement of the SITA standard strategy and for that reason we adopted it in our study designed in 1999. Later it became clear that the strategies performed slightly different. Two studies reported a slightly higher sensitivity for SITA standard in comparison with SITA fast [24], [25]; one study reported a higher sensitivity for SITA fast [26]. These differences – if any – are not relevant to the current study. More relevant to the current study is the finding that SITA fast seems to have a higher test-retest variability in areas with a reduced sensitivity in comparison with SITA standard [27]. This tentatively suggests that blindness reproducibility might be better in SITA standard and thus our criterion – three consecutive <0 dB readings – should be applicable to SITA standard as well.

In conclusion, current perimetric strategies share the inconvenient property that test-time increases in advanced glaucoma, while a smaller residual visual field has to be tested. A more clever customizing to what has to be tested than a default change to 10–2 testing should allow for an improved and uninterrupted long-term monitoring of glaucoma patients with standard automated perimetry.
